# Plasmacytoid Dendritic Cells Sequester High Prion Titres at Early Stages of Prion Infection

**DOI:** 10.1371/journal.ppat.1002538

**Published:** 2012-02-16

**Authors:** Rocio Castro-Seoane, Holger Hummerich, Trevor Sweeting, M. Howard Tattum, Jacqueline M. Linehan, Mar Fernandez de Marco, Sebastian Brandner, John Collinge, Peter-Christian Klöhn

**Affiliations:** 1 MRC Prion Unit and Department of Neurodegenerative Disease, UCL Institute of Neurology, London, United Kingdom; 2 Department of Statistical Science, University College London, London, United Kingdom; University of Alberta, Canada

## Abstract

In most transmissible spongiform encephalopathies prions accumulate in the lymphoreticular system (LRS) long before they are detectable in the central nervous system. While a considerable body of evidence showed that B lymphocytes and follicular dendritic cells play a major role in prion colonization of lymphoid organs, the contribution of various other cell types, including antigen-presenting cells, to the accumulation and the spread of prions in the LRS are not well understood. A comprehensive study to compare prion titers of candidate cell types has not been performed to date, mainly due to limitations in the scope of animal bioassays where prohibitively large numbers of mice would be required to obtain sufficiently accurate data. By taking advantage of quantitative *in vitro* prion determination and magnetic-activated cell sorting, we studied the kinetics of prion accumulation in various splenic cell types at early stages of prion infection. Robust estimates for infectious titers were obtained by statistical modelling using a generalized linear model. Whilst prions were detectable in B and T lymphocytes and in antigen-presenting cells like dendritic cells and macrophages, highest infectious titers were determined in two cell types that have previously not been associated with prion pathogenesis, plasmacytoid dendritic (pDC) and natural killer (NK) cells. At 30 days after infection, NK cells were more than twice, and pDCs about seven-fold, as infectious as lymphocytes respectively. This result was unexpected since, in accordance to previous reports prion protein, an obligate requirement for prion replication, was undetectable in pDCs. This underscores the importance of prion sequestration and dissemination by antigen-presenting cells which are among the first cells of the immune system to encounter pathogens. We furthermore report the first evidence for a release of prions from lymphocytes and DCs of scrapie-infected mice *ex vivo*, a process that is associated with a release of exosome-like membrane vesicles.

## Author Summary

Prions, rogue proteins that cause the fatal brain disease CJD in humans and BSE in cattle are not only found in the brain, but also in other tissues, particularly in lymphoid organs, long before they are detectable in the central nervous system. It is of great interest to better characterize how prions colonize the periphery after an infection and how they ultimately reach the brain, since such knowledge could help to develop treatments. By taking advantage of a technique called magnetic cell isolation we determined the infectious state of various immune cells, isolated from spleens of prion-infected mice. A high proportion of prions was detected in cells of the innate immune system, particularly in dendritic cells and natural killer cells. We furthermore found that small amounts of prions are released from infected cells, a finding which raises the question whether prions could spread in a similar manner to some viruses. These results suggest that prion-carrying immune cells that reside in the periphery may pose a major risk for the dissemination of prions, once they are mobilized, for example by an activation of the immune system.

## Introduction

Transmissible spongiform encephalopathies (TSE) or prion diseases are infectious and fatal degenerative disorders of the central nervous system including Creutzfeldt-Jakob disease (CJD) in humans, bovine spongiform encephalopathy (BSE) in cattle and scrapie in sheep and goats [1]. Prions, the infectious TSE agents, are thought to consist of abnormal forms of host-encoded cellular prion protein (PrP^c^) and to replicate in a self-perpetuating manner by recruitment of PrP^c^ [2], [3]. The disease-associated β-sheet rich conformer of PrP^c^, PrP^Sc^, is partially resistant to protease digestion and is argued to represent the infectious TSE agent [3]. More recently, protease-sensitive conformers of PrP^Sc^ have been identified that showed marked strain- and protease-dependent differences in their sensitivity to proteolysis [4]–[8].

In most TSEs, prions accumulate in the LRS long before they reach the brain. While prion accumulation in the LRS is not accompanied by any reported adverse effects, propagation of prions in the central nervous system inevitably leads to a rapid and progressive degeneration. Seminal work in the past two decades helped to identify critical cell types involved in prion colonization of the LRS [9]–[14]. Mobile hematopoietic as well as resident stromal cells play a crucial role in the pathogenesis of prion diseases [9], [15]–[17]. The adoptive transfer of bone marrow from wild type mice into PrP^0/0^ mice reconstituted the competence of the spleen to accumulate prions [18], [19]. However, scrapie histopathology in *Prnp* ^+/+^ neurografts was not observed under these conditions, implying that prion neuroinvasion is mediated by cells that cannot be reconstituted by bone marrow transfer [18]. There is good evidence that neuronal cells from the parasympathetic and sympathetic nervous system form a physical link between the LRS and the central nervous system [9], [10], [20]. The use of immuno-deficient mice greatly contributed to our understanding of peripheral prion colonization. The absence of clinical disease in B-cell deficient mice after intraperitoneal inoculation with prions was thought to indicate a direct role of B cells during neuroinvasion [11]. However, clear evidence suggests that the maintenance and differentiation of follicular dendritic cells (FDC) and other stromal cells by B cell-dependent lymphotoxin β receptor (LTβR) signalling may best explain the role of B cells during prion colonization in the LRS and during neuroinvasion [12]–[14], [21], [22]. Whilst FDCs were previously considered the prime candidate for the site of prion replication in the LRS, observations of an unimpeded neuroinvasion in absence of FDCs in mice deficient in TNFα signalling [22] suggested that other stromal cells may also be prion-replication-competent. A recently identified stromal cell type in granulomas, presumably mesenchymal or fibroblastic reticular cells, that are dependent on LTβR signalling has been shown to promote prion replication in absence of FDCs [23].

Due to their pivotal role in immune defence against pathogens and their migratory properties, antigen-presenting cells like dendritic cells (DCs) and macrophages are likely candidates for the dissemination of prions. DCs were suggested as mobile carriers for prions from the gut to the LRS after intra-intestinal injection of scrapie-associated fibrils [24]. Rag-1^−/−^ mice injected intravenously with infected DCs succumbed to scrapie [25], demonstrating that, at least under these experimental conditions, DCs can transmit disease from the periphery to the CNS without prion accumulation in the LRS. Prion infectivity was also found to be associated with macrophages. Early fractionation experiments of splenic cell types based on differences in their buoyant densities identified prions in a macrophage-rich fraction, but an enrichment of this fraction failed to enhance infectivity [26]. Immuno-electron microscopic studies identified PrP deposits associated with tingible body macrophages [27]. The temporal depletion of macrophages *in vivo* led to increased PrP^Sc^ levels in the spleen [28] or Peyer's patches [29], suggesting a role of macrophages in the clearance of infectivity. After oral infection, prions were detected in Peyer's patches of the gut-associated lymphoid tissue in different animal species [30]–[33]. The transport of prions across the intestinal epithelium is believed to be mediated by intestinal membranous or microfold cells (M cells) [34], [35].

In contrast to our understanding of molecular factors that promote prion replication in lymphatic organs, the contribution of mobile cells of hematopoietic origin to prion dissemination in the LRS is not well characterized. A comprehensive study to determine the infectious state of candidate cell types during early stages of pathogenesis has not been performed to date due to the prohibitively large number of animals required. The recently established quantitative *in vitro* infectivity assay, the Scrapie Cell Assay (SCA) [36], [37] now renders such experiments feasible. We here established a procedure to isolate various splenic cell types, including B and T lymphocytes, dendritic cells (DC), the DC subtype plasmacytoid DCs (pDC), macrophages and natural killer cells by magnetic-activated cell sorting (MACS) followed by the determination of infectious titers by SCA. Our results characterize the time-dependent accumulation of prions in splenic cell types of 129Sv×C57BL/6 mice during the first four weeks after inoculation with mouse prions, a time interval that yielded maximal prion titers in the spleen, and demonstrate that pDCs and NK cells, two cell types that have previously not been associated with prion dissemination, are highly infected.

A reliable determination of prion titers is fundamental to the study of prion diseases where differences in titers may be critical to assess the efficacy of therapeutic interventions. Where the size of experimental groups in animal bioassays is limited by ethical and economic considerations, *in vitro* determination of prion titers can overcome these limitations and allow rapid accurate bioassay of large numbers of samples [38]. The estimation of statistically robust titers in this study was obtained by statistical modelling using the generalized linear model [39] along with maximum likelihood estimation.

Molecular events that lead to the dissemination and neuroinvasion of prions are unknown. *In vitro*, several routes for the transmission of prions, like direct cell-to-cell contact [40], prion transmission via membrane nanotubes [41], [42] and the release of prions via exosomes [43] have been suggested. Exosomes, small membrane vesicles secreted by most hematopoietic cells are present *in vivo* in germinal centres [44] and body fluids [45]–[51]. We here present the first evidence that MACS-isolated lymphocytes and DCs from prion-infected mice secrete prions into the cell supernatant when cultured *ex vivo*, a process that was associated with the secretion of exosome-like particles. We furthermore present experimental evidence that prions are physically associated with exosome-like particles.

## Results

### Isolation of splenic cell types by magnetic-activated cell sorting

**Figure 1:**
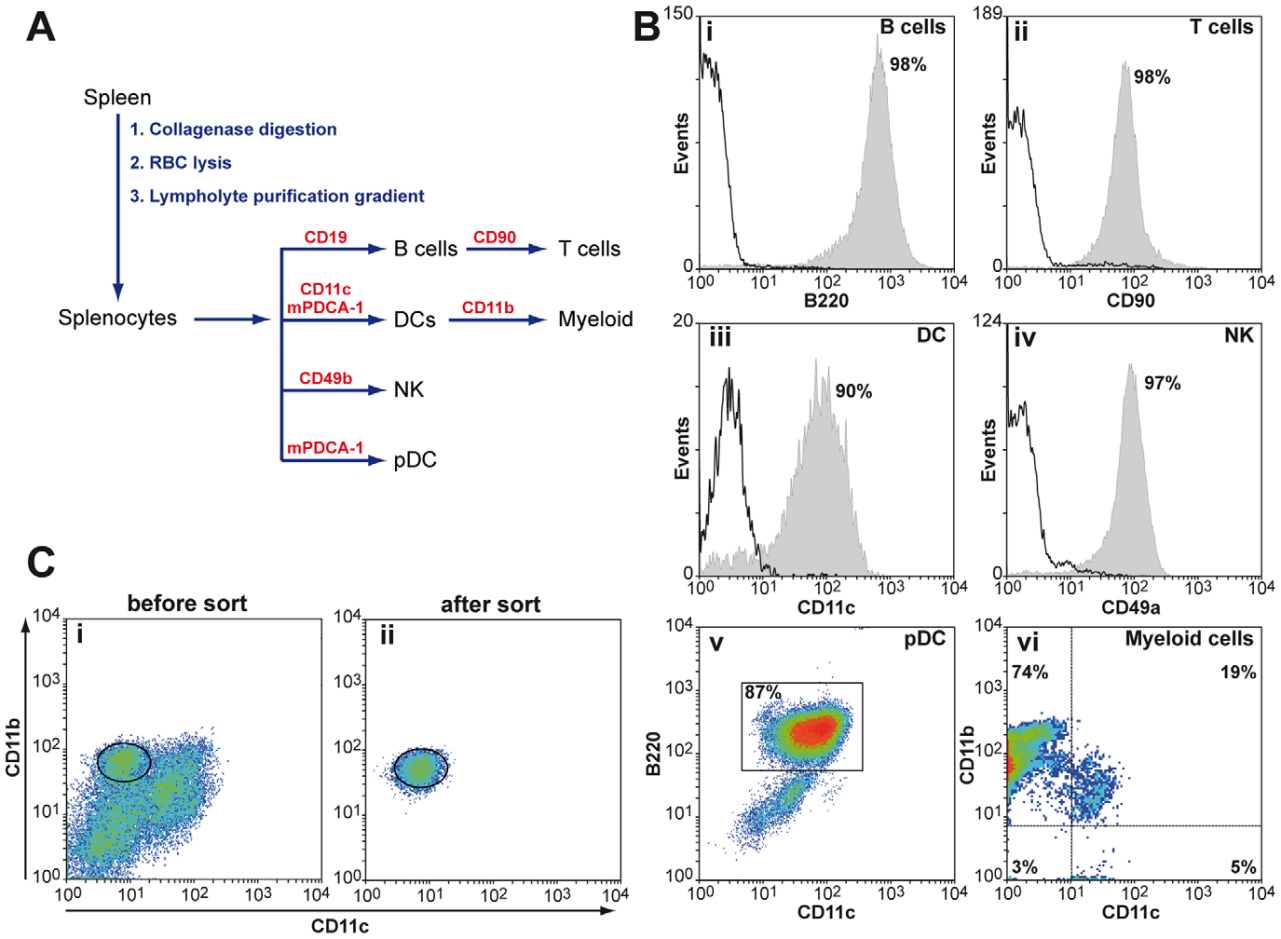
Isolation of splenic cell types by magnetic-activated cell sorting. **A**: Schematic representation for the isolation of specific splenic cell types from mice. Splenocytes were released by repeated collagenase digestion from freshly dissected spleens, followed by removal of erythrocytes and purification of splenocytes on Lympholyte M gradients. Splenic cell types are isolated by positive selection with magnetic beads coated with cell type-specific mAbs as specified. **B**: The purities of MACS-isolated cells were analysed by FACS using cell-type specific mAbs and isotype controls as specified in Materials and Methods. One representative out of three experiments is shown. (Bv) CD11^low^ B220^+^ pDCs, isolated with murine plasmacytoid dendritic antigen-1 (mPDCA-1) showed a purity of about 90% in three independent experiments. (Bvi) The macrophage population, isolated with CD11b microbeads after depletion of CD11c^+^ cells was contaminated with CD11c^+^ CD11b^+^ mDCs. Macrophages were therefore isolated by FACS instead (**C**). **C**: Splenocytes labelled with mAbs against anti-CD11b (M1/70) and anti-CD11c (HL3) were isolated by FACS using a DAKO cell sorter.

The recent establishment of the SCA, a highly sensitive *in vitro* infectivity assay [36], [52] enables us to examine the kinetics of prion accumulation in splenic cell types at early stages of prion pathogenesis in an unprecedented manner. We used MACS to isolate splenic cell types from a mixed population of splenocytes with purities from about 87% (pDC) to more than 95% (NK, B and T cells) (Figure 1) and then determined infectious titers. MACS isolation is an excellent tool for isolating rare cell types from large pools of mixed cell populations at reasonable processing times. For the isolation of DCs, for instance, 6×10^8^ splenocytes were processed in about an hour with an average yield of 4% (2.4×10^7^), as compared to hundred-fold lower rates using fluorescence-activated cell sorting (FACS). Where a surface marker for specific cell types was expressed at low levels, or on more than one cell type, the isolation procedure was adapted accordingly. Three DC subtypes can be distinguished by means of their surface markers: CD11^+^ CD11b^+^ myeloid DCs (mDC), CD11^+^ CD8α^+^ lymphoid DCs (lDC) and CD11^low^ B220^+^ plasmacytoid DCs (pDC). Since pDCs express low levels of CD11 which may compromise their quantitative isolation with CD11 microbeads we used microbeads coated with monoclonal antibodies (mAbs) against murine plasmacytoid dendritic cell antigen-1 (mPDCA-1), a protein that is specifically expressed in mouse pDCs [53]. Accordingly, panDCs were isolated with a 1∶1 mixture of CD11c and mPDCA-1 microbeads. CD11b, a surface marker for myeloid cells that is broadly utilized for the isolation of macrophages is also expressed on CD11c^+^CD11b^+^ mDCs. To avoid an enrichment of mDCs in the macrophage cell population we isolated DCs prior to macrophages. However, despite the depletion of DCs by positive selection with CD11c beads, the macrophage fraction contained a substantial amount of CD11^+^ CD11b^+^ myeloid DC contaminants (Figure 1B). This prompted us to use fluorescence-activated cell sorting to separate CD11b^+^ macrophages from myeloid DC contaminants (Figure 1C).

### Quantification of prion titers using a generalized linear model

**Figure 2:**
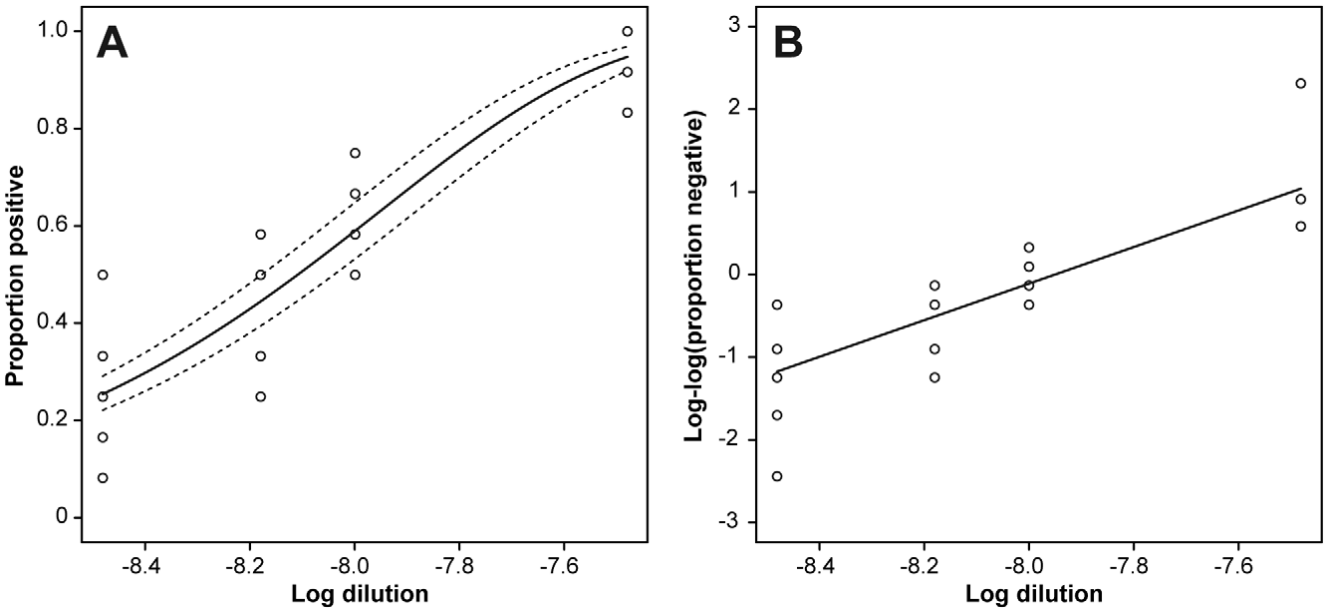
Regression analysis of SCEPA endpoint titration assay using GLM. For the estimation of prion titers by SCEPA, prion-susceptible cells were infected with serially diluted RML I6200 brain homogenate and the proportions of scrapie-positive wells were analysed using a GLM with binomial family and complementary log-log link. **A**: Observed and estimated proportions of scrapie-positive wells with 95% confidence intervals. **B**: Linearized link-transformed proportions of scrapie-positive wells. Here the four zero values at dilution 10^−8^ were replaced by 0.5 in order to plot the observed loglog values. Data represent eight technical assay repeats of serially diluted RML I6200 brain homogenate.

The SCA is based on the detection of single PrP^Sc^-positive cells that are formed by *de-novo* prion propagation after infection with prion-containing samples [36]. A more sensitive version of the assay, the Scrapie cell assay at endpoint format (SCEPA) exploits the observation that the sensitivity for prion detection can be significantly improved by varying the cell splitting ratio [36], [52]. Using SCEPA, infectious titers are determined at limiting dilutions of prion-containing samples [54]. Whilst in animal bioassays prion titers are commonly expressed as simple median lethal doses (LD_50_ units) and estimated by non-parametric analysis [55], the average number of infectious units in the SCEPA, here termed tissue culture infectious units (TCIU) can be estimated by assuming that the number of PrP^Sc^-positive cells at a given dilution follows a Poisson distribution [54]. Inherent to limiting dilution assays, however, the error variance may not be constant over the studied range of dilutions (Figure 2) and thus may lead to inflated errors when ordinary regression analysis is used. To address this problem we here established a generalized linear model (GLM) [39] for the estimation of infectious titers. GLMs overcome restrictions of ordinary linear regression models which are limited to normally distributed response variables with constant variance and unify a wide range of probability distributions, including normal, binomial, Poisson and gamma by the use of a common method for computing maximum likelihood estimates (Text S1). The GLM framework can be equally applied to estimate infectious titers from animal bioassays, where repeated measurements are available.

We first examined whether the number of PrP^Sc^-positive cells in the SCEPA follows a Poisson distribution. At limiting dilutions of infectivity the number of positive wells in 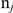 independent infections at the *j*th dilution follows a binomial distribution with parameters 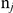 and 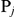 where 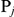 is the proportion of positive wells. If the number of prion-infected cells is assumed to have a Poisson distribution then the proportion of negative wells 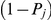 is equal to 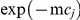, where *m* is the mean number of infectious units per volume and 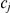 the dilution. A complementary log-log transformation converts this equation to
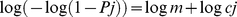
(1)Thus if the number of scrapie-infected cells follows a Poisson distribution then a complementary log-log transformation is linear with a slope of one. To check this hypothesis we prepared multiple dilution series of brain homogenate, infected susceptible cells and determined the number of negative wells. RML brain homogenate I6200 was serially diluted 1∶3 from 10^−7^ to 10^−9^ and cell layers of 12 wells per dilution were infected using eight technical repeats per dilution. An initial linear regression analysis resulted in an estimated slope of 1.06±0.20 (Figure S1), in agreement with the assumption of an underlying Poisson distribution for the number of infected cells. This prompted us to calculate infectious titers using a GLM, for which a flexible iterative method for maximum likelihood estimation is available [39]. Using the GLM approach we can fit the proportions of positive wells with the regression model
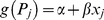
(2)where *g* is a link function, here the complementary log-log transformation, *α* the log mean infectious units, *β* the regression slope and 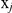 the log dose. An estimated value for *β* of 0.960±0.096 is consistent with the hypothesis of an underlying Poisson distribution for the number of infected cells and the model provides a good fit to the data (Figure 2 and Table S1). GLM regression yielded an estimated titer of 8.63±0.03 logTCIU/g brain for eight technical repeats of serially diluted RML I6200 brain homogenate.

### Determination of the sensitivity of SCEPA

To determine the relative sensitivity of SCEPA against the mouse bioassay we performed endpoint titrations with RML I6200 in parallel experiments. Titers from eight independent *in vitro* assays were highly reproducible and yielded an estimated titer of 8.71±0.04 logTCIU/g brain by GLM (Table 1). On mouse bioassay, infectious titers were about half a log higher, albeit with a higher variance.

**Table 1:** Sensitivity for prion detection of SCEPA and mouse bioassay.

RML input[LD_50_ units]	SCEPA*	Tga20 mouse bioassay 1		Tga20 mouse bioassay 2	
	PrP^Sc^-positive/total wells	sick/total	Inc. time, days ± SD	sick/total	Inc. time, days ± SD
**6×10^3^**	ND	5/5	75.8±1.2	ND	ND
**6×10^2^**	ND	5/5	83.2±3.6	5/5	86.2±2.8
**6×10^1^**	24/24	6/6	106.5±6.4	5/5	112.6±5.9
**6×10^0^**	15/24	3/4	110.3±4.0	3/5	122.3±6.3
**6×10^−1^**	2/24	1/5	116	1/5	104
**6×10^−2^**	0/24	2/6	159.5±1.0	1/6	136
**6×10^−3^**	ND	0/6	>200	0/6	>200
**Infectious titers**	*Log TCIU/g ± SE*	*Log LD_50_/g ± SE*		*Log LD_50_/g ± SE*	
Spearman-Karber†	8.70±0.19	9.31±0.42		8.99±0.41	
GLM$	8.71±0.04	9.02±0.23‡			

*: The ratio between PrP^Sc^-positive and total wells is shown for one representative out of eight independent experiments.

**†:** Infectious titers were calculated with the Spearman-Karber formula [55] and expressed as tissue culture infectious units (TCIU)/g brain for SCEPA and LD_50_ units/g brain for mouse bioassay, respectively.

$ Infectious titers were calculated using a GLM with binomial family complementary log-log link and expressed as mean log TCIU/g brain ± SE of 8 independent experiments for SCEPA and mean log LD50 units/g ± SE for two independent bioassays.

**‡:** Infectious titers were estimated for the combined two bioassays using a GLM regression with complementary log-log link function and expressed as log LD50 units/g brain.

The sensitivity for prion detection of SCEPA and mouse bioassay was determined by endpoint titration using RML mouse brain homogenate I6200. Aliquots of I6200 (10% (w/v), 9.3 log LD_50_ units/g brain [7]) were serially diluted 1∶10 into uninfected CD1 brain homogenate (10% w/v) in a range between 10^−4^ and 10^−10^. For mouse bioassay, groups of six Tga20 mice were inoculated intracerebrally with 30 µl of 1% (w/v) RML homogenates and attack rates and scrapie incubation times (Inc. time) were determined. In parallel experiments brain homogenates were diluted 1∶1000 into OFCS and cell layers of highly prion susceptible N_2_aPK1-2 cells were infected with 300 µl aliquots. The input of prion infectivity for bioassay and SCEPA is expressed as mouse ic LD_50_ units. A 10^−7^ dilution of I6200 corresponds to 200 LD_50_ units/ml or 6 LD_50_ units per 30 µl inoculum for the mouse bioassay and 60 LD_50_ units per 300 µl per well for SCEPA, respectively. Infectious titers for SCEPA, expressed as TCIU/g brain represent mean values ± SE of 8 independent experiments. For mouse bioassay, two independent experiments are shown and titers are expressed as LD_50_/g brain ± SE.

In summary, the SCEPA outperforms the mouse bioassay in terms of statistical robustness, low cost and speed, while the somewhat lower sensitivity may be addressed by increasing the number of technical repeats. Of note, N_2_a-derived cells are permissive to prion strains RML and 22L only, but not to other mouse-adapted prion strains like Me-7, 22A and 301C. Prion-susceptible cell lines with a broader susceptibility for mouse-adapted prion strains have been identified recently [52], [56] and can be used instead of N_2_a cells. It should be noted, though, that the sensitivity of the SCEPA is cell-type dependent.

### Validation of a cell homogenization method

The dispersion state of prion-infected homogenates is a critical parameter where limiting dilutions are used to determine infectious titers. An increase in dispersion of an infected homogenate will result in an apparent increase of infectivity at limiting dilutions. We therefore sought to establish a standardized method for tissue and cell homogenization. Homogenization by shear force with needles, a method broadly used to generate tissue homogenates for prion titer determination failed to homogenize splenocytes as indicated by a high percentage of Trypan blue-negative viable cells. We therefore tested two alternative homogenization methods, sonication and ribolyzation, both of which lead to complete cell homogenization. Infectious titers of B lymphocytes and pDCs were determined using the mouse bioassay and SCEPA in parallel experiments (Table 2). Infectious titers of B cells determined by mouse bioassay at 30 dpi were in agreement with previously published data [57]. No significant differences in infectious titers were observed between the two homogenization methods for both cell types using SCEPA and bioassay, respectively, except for pDCs where ribolyzation resulted in significantly higher prion titers as compared to sonication when assayed by SCEPA (see Table 1). An assay-dependent difference in titers for SCEPA and bioassay of about one log was determined, which accounts for the lower sensitivity of the *in vitro* assay. Remarkably, infectious titers of pDCs exceeded those of B lymphocytes by more than half a log, irrespective of the assay and homogenization method used. For all subsequent experiments ribolyzation was used as a standard homogenization method to exclude the risk of cross-contamination during sonication of prion-infected samples.

**Table 2:** Infectious titers of MACS-isolated cells after homogenization by sonication and ribolyzation.

(A) SONICATION				
	Cell number	SCEPA	Mouse bioassay	
Cell Types	equivalents‡	PrP^Sc^-positive/total wells	Attack rate	Incub. time (d) ± SD
**pDC**	6×10^3^	12/12	5/5	89±1
	6×10^2^	5/12	5/5	117±9
	6×10^1^	1/12	2/6	131±17
	6×10^0^	0/12	0/6	>200
**control** #	6×10^3^	0/12	0/6	>200
**Infectious titers**		***Log TCIU/10^6^ cells ± SE***	***Log LD_50_/10^6^ cells ± SE***	
Spearman-Karber†		3.21±0.08	4.06±0.25	
GLM$		3.13±0.04	-	
**B cells**	6×10^4^	12/12	5/5	92±4
	6×10^3^	8/12	6/6	99±2
	6×10^2^	2/12	5/6	124±7
	6×10^1^	0/12	1/6	171
**control** #	6×10^4^	0/12	0/4	>200
**Infectious titers**		***Log TCIU/10^6^ cells ± SE***	***Log LD_50_/10^6^ cells ± SE***	
Spearman-Karber†		2.61±0.19	3.72±0.30	
GLM$		2.53±0.03	-	

(B) RIBOLYZATION				
	Cell number	SCEPA	Mouse bioassay	
Cell Types	equivalents‡	PrP^Sc^-positive/total wells	Attack rate	Incub. time (d) ± SD
**pDC**	6×10^3^	12/12	4/4	85±1
	6×10^2^	6/12	5/6	105±5
	6×10^1^	1/12	4/6	108±8
	6×10^0^	0/12	0/6	>200
**control** #	6×10^3^	0/12	0/4	>200
**Infectious titers**		***Log TCIU/10^6^ cells ± SE***	***Log LD_50_/10^6^ cells ± SE***	
Spearman-Karber†		3.31±0.15	4.22±0.33	
GLM$		3.24±0.04 (p<0.05)*	-	
**B cells**	6×10^4^	12/12	6/6	88±3
	6×10^3^	9/12	5/5	100±5
	6×10^2^	2/12	5/5	107±12
	6×10^1^	0/12	1/5	174
**control** #	6×10^4^	0/12	0/3	>200
**Infectious titers**		***Log TCIU/10^6^ cells ± SE***	***Log LD_50_/10^6^ cells ± SE***	
Spearman-Karber†		2.70±0.15	3.92±0.29	
GLM$		2.53±0.03	-	

**‡:** Inputs of infectious cell homogenates are expressed as cell number equivalents. Aliquots of 30 µl were inoculated i.c. into groups of six Tga20 mice for mouse bioassay and 300 µl aliquots were layered onto prion-susceptible cells per well for SCEPA, respectively.

**†:** Infectious titers were calculated according to the Spearman-Karber method [55] and are expressed as log LD_50_/g ± SE for bioassay and log TCIU/g ± SE for SCEPA.

$ Infectious titers were calculated using a GLM with binomial family complementary log-log link and expressed as mean log TCIU/g ± SE of two independent experiments with six technical repeats each.

# Controls represent MACS-isolated pDCs and B lymphocytes from spleens 129/Sv×C57BL/6 mice inoculated with 1% (w/v) uninfected CD1 brain homogenates and sacrificed at 30 dpi.

*: Level of significance for maximum likelihood estimates (GLM) between infectious titers of ribolyzed versus sonicated pDCs as determined by SCEPA.

Four 129/Sv×C57BL/6 mice were inoculated i.p. with 100 µl of 1% (w/v) RML and 1% (w/v) uninfected CD1 brain homogenate (control), respectively. At 30 d.p.i spleens were dissected and pDCs and B cells isolated by MACS according to Figure 1. Aliquots of 1×10^7^ cells/ml OFCS, supplemented with protease inhibitors were homogenized by sonication *(A)* or ribolyzation *(B)* according to Materials and Methods. To determine infectious titers the cell homogenates were serially diluted 1∶10 and inoculated intracerebrally into Tga20 mice or transferred onto layers of susceptible PK1-2 cells in parallel experiments. Infectious titers were determined by non-parametric statistical analysis for bioassay (Spearman and Karber) and GLM for SCEPA and expressed as log LD50 units/10^6^ cells and log TCIU/10^6^ cells, respectively. A 10^−2^ dilution of cell homogenates corresponds to 2×10^5^ cell equivalents/ml or 6×10^3^ cell equivalents per 30 µl inoculum for mouse bioassay and 6×10^4^ cell equivalents per 300 µl per well for SCEPA, respectively. Infectious titers represent log mean values ± SE of six independent experiments for SCEPA and log mean values ± SE of a single experiment for mouse bioassay.

### Prion accumulation in the lymphoreticular system at early stages of prion disease

**Figure 3:**
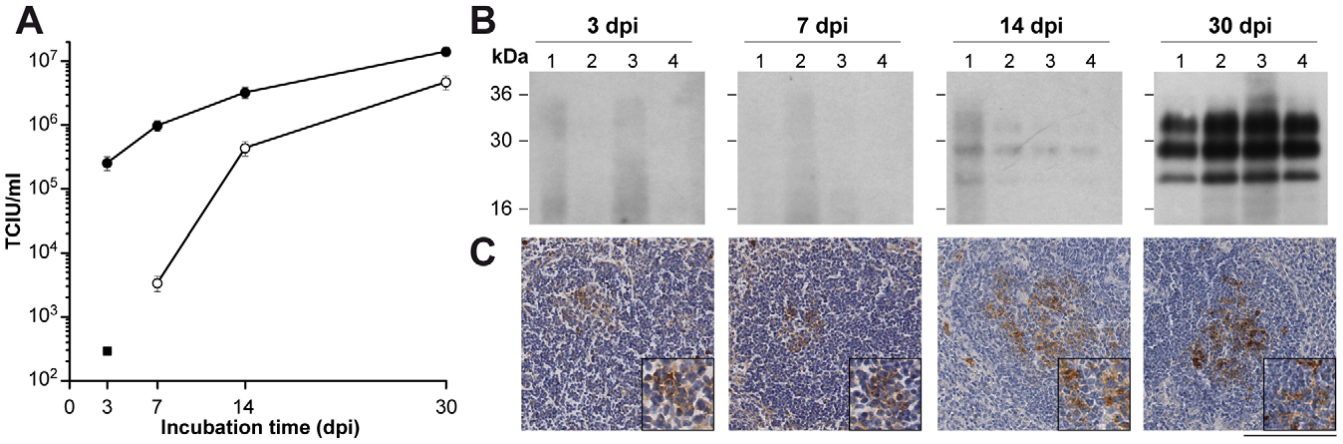
High splenic prion titers at early stages after prion infection. Groups of four 129/Sv×C57BL/6 and *Prnp* ^0/0^ mice were inoculated i.p. with 100 µl 1% (w/v) RML I6200 (9.3 log LD_50_ units/g brain). At various time points after inoculation spleens and mesenteric lymph nodes were dissected and prion titers and PrP^Sc^ levels determined. (**A**) Infectious titers of spleens (closed circles) and mesenteric lymph nodes (open circles) of 129/Sv×C57BL/6 mice. To account for residual inoculum infectious titers of spleens of RML-inoculated Prnp^0/0^ mice (closed square) were determined. Prion titers were estimated by a GLM with binomial family complementary log-log link. Data represent mean infectious titers ± SD of four repeats. (**B**) Spleen homogenates were analyzed for levels of PK-resistant PrP (PrP^Sc^) by Western blotting after NaPTA precipitation as described in Materials and Methods. (**C**) Detection of PrP^Sc^-positive deposits in spleens of prion-infected 129/Sv×C57BL/6 mice. At 3 dpi less than 5% of the total number of follicles was weakly PrP^Sc^-positive. An increase in the number of immunopositive follicles, but overall a weak immunostaining for abnormal PrP was observed at 7 dpi. At 14 dpi, the number of positive follicles was similar, but the staining intensity increased to ‘moderate’ in two animals. At 30 dpi 90% of lymphoid follicles showed moderate or strong labeling. The scale bar corresponds to 100 µm (20 µm in the inserts).

To assess the rate of prion accumulation in the lymphoreticular system at early stages of disease we first determined prion titers in spleen tissue and mesenteric lymph nodes after intraperitoneal inoculation of 129 Sv×C57BL/6 mice and *Prnp* ^0/0^ mice with 1% (w/v) RML I6200. Prion titers of more than 5 log TCIU/g spleen tissue were determined at stages as early as 3 dpi (Figure 3A). In contrast, in *Prnp* ^0/0^ mice, prion titers which are due to residual inoculum [58] were about a thousand-fold lower at the same incubation time, suggesting an exceptional rate of prion replication in wild-type mice and/or differences in the efficiency of trapping prions. Prion titers in mesenteric lymph nodes were significantly lower as compared to spleen titers in accordance to previous reports [59]. At 30 dpi infectious titers in spleens and mesenteric lymph nodes reached 6.63±0.07 log TCIU/g tissue and 6.15±0.10 log TCIU/g tissue, respectively. Given the somewhat lower sensitivity of SCEPA splenic prion titers are in agreement with previously published bioassay data using the same mouse strain, infectious dose and inoculation route (∼7 ic LD50 units/g spleen) [60].

To test whether the fast splenic prion accumulation at early stages of disease is in accord with the detection of abnormal PrP deposits in lymphoid follicles we examined PrP accumulation by PrP immunohistochemistry at 3, 7, 14 and 30 dpi (Figure 3C and Table S2). Deposits of abnormal PrP were detected at low intensity and frequency in follicles at 3 dpi, and both, the number of positive follicles and the PrP intensity increased significantly over the course of the incubation (Fig. 3 and Table S2). At 30 dpi 90% of follicles were PrP^Sc^-positive (Table S2). Abnormal PrP could not be detected in lymphoid follicles of Prnp^−/−^ mice at 3 dpi and 7 dpi (data not shown). Furthermore, PrP^Sc^ could not be detected by Western blotting in spleen tissue prior to 14 dpi (Figure 3B).

### The kinetics of prion accumulation in splenic cell types

We next determined infectious titers of MACS-isolated cells in a time-dependent manner to assess the propensity of distinct splenic cell types to accumulate prions. Whilst prions were detectable in all cell types, including B and T lymphocytes, DCs, NKT cells and macrophages, highest infectious titers were determined in two cell types that have previously not been associated with prion pathogenesis: pDCs and NK cells (Table 3). At 30 dpi mean infectious titers of NK cells were more than two-fold higher than those of lymphocytes, whereas titers of pDC exceeded those of lymphocytes by a factor of seven. Prion titers were significantly higher in pan-DCs as compared to those of lymphocytes (Table 3), in agreement with previous studies [25]. Data were replotted for 30 dpi values in Figure S2. A relative increase of infectious titers for all cell types by 30–50% from 3 dpi to 30 dpi correlated with an increase of splenic prion titers during the same time interval (Figure 3). We next investigated whether infectious inoculum was detectable in splenic cell types of *Prnp* ^0/0^ mice, i.e. in absence of prion replication (Table S3). At 3 dpi, infectious titers of macrophages (0.47±0.17 TCIU/10^6^ cells) and pan DCs (0.23±0.09 TCIU/10^6^ cells) were about five to ten times higher than those of lymphocytes (0.06±0.06 TCIU/10^6^ cells), indicating that infectivity was primarily associated with antigen-presenting cells.

**Table 3:** Time-dependent accumulation of prion infectivity in isolated splenic cell types.

	Infectious titers (TCIU/10^6^ cells)					
Cell types	3 dpi	7 dpi	14 dpi (95% conf.int)#		30 dpi (95% conf.int)#	
Splenocytes	11	30	265±64	(147, 480)	544±124	(311, 952)
DC	23	62±14	265±64	(147, 480)	677±87	(518, 885)
pDC	41	106	370±85	(211, 650)	1798±298	(1252, 2579)
Myeloid cells‡	1	37±9	127±34	(67, 244)	472±57	(365, 609)
Macrophages†		-	-		243±49	(345, 342)
B cells	9	26±6	130±35	(68, 249)	262±28	(210, 328)
T cells	9	25±2	135±36	(71, 260)	208±28	(156, 279)
NK(T) cells*	-	-	183		721±120	(491, 1059)
pDC (blood)	-	-	-		<5	
DC (blood)	-	-	-		<5	
PBC (blood)					<5	

**‡:** Myeloid cells were isolated with CD11b magnetic beads after partial depletion of DCs and contain CD11c^−^ CD11b^+^ macrophages and CD11c^+^ CD11b^+^ mDCs (see Figure 1B).

**†:** CD11b^+^ macrophages devoid of mDCs contaminants were isolated by FACS (see Figure 1C).

# Infectious titers are represented as mean values ± SE, and as lower and upper limits of 95% confidence intervals (conf.int).

Groups of ten 129/Sv×C57BL/6 mice, inoculated i.p. with 100 µl aliquots of 1% (w/v) RML brain homogenate I6200 were culled at various time points after inoculation as specified above and splenocytes and splenic cell types were serially isolated by MACS after Collagenase digestion and Lympholyte purification according to Figure 1. Infectious titers were determined by SCEPA using a GLM as specified above. Mean values ± SE and 95% confidence intervals for at least three independent experiments are shown at 14 and 30 dpi. Data from a single experiment are shown where no SE is reported. The detection limit of the assay for splenic cell types was 0.15 TCIU/Mio, for MACS-isolated cells from whole blood 5 TCIU/Mio.

The high prion titers in pDCs (Table 3) raise the question whether pDCs replicate prions. Of note, the presence of PrP^c^, a pre-requisite for prion replication was reportedly undetectable in pDCs [61], [62]. To exclude mouse strain-dependent differences in PrP^c^ expression levels we labeled MACS-isolated pDCs from uninoculated 129 Sv×C57BL/6 mice with mAb ICSM18 against PrP^c^ (Figure S3). In agreement with previous reports [61] PrP^c^ expression in pDCs was undetectable, thus rendering pDCs unlikely candidate cells for prion replication.

Prions have been detected at extremely low titers in blood of rodents at presymptomatic and symptomatic stages and were associated with buffy coat and plasma fractions [63]–[68]. In a previous report, infectivity was not detected in peripheral blood leukocytes in 129Sv×C57BL/6 mice at early stages of disease regardless of relatively high titers in B and T lymphocytes of the spleen [57]. In marked contrast to lymphocytes, DCs have a restricted capacity for recirculation, a propensity that may protect the host by retaining a high density of peptide-MHC complexes for improved antigen presentation [69]. Given the high infectious titers of DCs we scrutinized the possibility of prion spread by recirculation. To determine whether prion infectivity is associated with pDCs in blood we isolated pDCs from EDTA-treated whole blood. However, no infectivity was associated with pDCs, lymphocytes and DCs from blood at 30 dpi under our experimental conditions (Table 3, and Materials and Methods).

It has been broadly acknowledged that prions do not mount a humoral immune response in the host [70]–[72]. However, a recent study showed an abnormal germinal center reaction in the spleen of scrapie-infected mice which was associated with increased maturation and numbers of B lymphocytes and hypertrophy of FDC dendrites at 70 dpi and endstage [73]. A further report showed variations in the number of CD21^+^ B cells in lymph nodes of prion-infected sheep [74]. We therefore examined whether B cells or DCs from 129Sv×C57BL/6 mice were activated at preclinical stages. However, the proportions of marginal zone B cells (CD21^hi^ CD23^−^) and follicular B cells (CD21^int^ CD23^hi^) in scrapie-infected versus age-matched mock-infected mice were unchanged at 80 and 100 dpi and no activation of DCs was evident at preclinical stages of disease (Figures S4 and S5).

### Ex-vivo release of prions from scrapie-infected splenic cells

**Figure 4:**
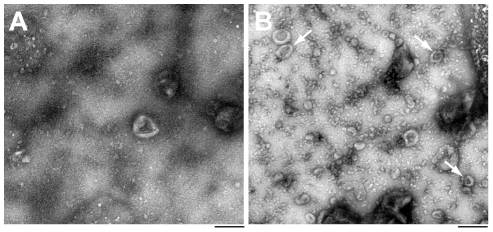
Exosomes are released from scrapie-infected B cells *ex vivo*. Spleens were dissected from 129/Sv×C57BL/6 mice 30 days after i.p. inoculation with 1% (w/v) RML I6200. MACS-isolated B lymphocytes were cultured under passive leakage (A) and basal (B) conditions essentially as described in Table 4 and tissue culture supernatants were isolated by sequential centrifugation (Materials and Methods). After centrifugation at 100,000× g for 2 h pellets were resuspended in PBS, absorbed onto carbon-coated grids and negatively stained with 1% uranyl acetate. Cup-shaped exosome-like membrane particles of different sizes (see arrows) are shown in Figure 1B. Twenty randomly recorded images (surface area: 2.82 µm^2^) from each condition were counted and the number of exosome-like particles (1.7±1.2 (A) and 22.8±6.5 (B) per surface area, p≪0.001) determined in a blinded manner. Scale bar: 0.2 µm.

The molecular underpinnings of prion dissemination *in vivo* are unknown. Several routes for the horizontal transmission of prions have been suggested, including direct cell-to-cell contact [40], the release of prions via exosomes [43], [75], [76], and prion transmission via membrane nanotubes [41], [42]. The *in vivo* relevance of these processes has not been demonstrated and poses major experimental challenges. Exosomes are small vesicles of endosomal origin that were detected on the surface of FDCs *in vivo* [44]. Since hematopoietic cells like reticulocytes, mast cells, B and T lymphocytes, DCs and macrophages release exosomes [77]–[81], we investigated whether prions are released from scrapie-infected splenic cells *ex vivo*. Freshly isolated B and T lymphocytes and DCs from scrapie-infected mice were cultured for 38 h and culture supernatants were sequentially centrifuged according to exosome isolation protocols [82]–[84] (see Material and Methods). After ultracentrifugation, pellets were resuspended in medium and prion infectivity was determined by SCEPA. As evident from preliminary experiments, splenic cells, particularly B and T lymphocytes, showed a limited viability *ex vivo* which may bias the determination of prion secretion where prions are released by passive leakage from necrotic cells. To account for the contribution of passive leakage of prions from dead cells we cultured isolated cells at atmospheric CO_2_ at 37°C in parallel experiments, a treatment that led to rapid necrosis of B and T lymphocytes (Table 4). Where exposure of cells to atmospheric CO_2_ did not suffice to trigger rapid necrosis as in the case of DCs, we added low concentration of Triton X-100 (0.01% final) to the culture medium to permeabilize cells. A more than 30-fold increase of infectivity was detected in supernatants of B lymphocytes and DCs under basal conditions as compared to passive release controls, demonstrating that prions are released from scrapie-infected cells (Table 4). Supplementation of medium with IL-4, a treatment that leads to activation of lymphocytes improved the viability of B cells with a moderate increase in prion titers of supernatants. Incubations of isolated B lymphocytes with bacterial lipopolysaccharide (LPS) which differentiates B cells into plasmablasts lead to a significant decrease of prion release. This may indicate that the pool of secretable prions is reduced by an increased proteolytic activity of DCs under these conditions [85]. The titers of released prions constitute about 1–3% of the cellular infectivity of B lymphocytes and DCs. Similar data have been reported for the *in vitro* release of PrP^Sc^ from cell lines [86]. To examine whether prion secretion in B lymphocytes is associated with a release of exosomes we pelleted cell culture supernatant from cells cultured in basal medium or atmospheric CO_2_ by ultracentrifugation and resuspended pellets in PBS and absorbed small aliquots onto EM grids for microscopic analysis (Figure 4). The number of exosomes, identifiable by their typical cup-shaped morphology [48], [87], [88], ranging in diameter from 20 to 100 nm [89], under basal conditions exceeded the number of exosomes during passive leakage by a factor of more than fifteen (1.4±1.2 versus 22.8±6.5 per count area, p≪0.001, Figure 4). Microparticles, shed by apoptotic or stimulated cells ranging from 200 to 1000 nm in diameter [89]–[92] were detected infrequently under our experimental conditions with rates below 0.4 microparticles per count area with no significant difference between basal medium and passive leakage control. The minute amounts of released exosomes under our *ex vivo* culture conditions did not allow the detection of exosome-associated proteins by Western blotting. To investigate whether prions are physically associated with exosomes we immunoisolated prions from concentrated cell supernatants of B cell and splenocyte cultures with antibodies against exosome markers CD81 [93]–[95] and Rab 5B [96] (Material and Methods). A more than 4-fold (8.5 TCIU) and 2-fold (3.8 TCIU) enrichment of prions was determined after immunoisolation with anti-Rab 5B and anti-CD81, respectively, as compared to isotype controls (1.8 TCIU). A four-fold enrichment of prions from splenocyte cultures was determined after immunoisolation with anti-CD81 (35 TCIU) as compared to an isotype control (8.5 TCIU).

**Table 4:** Prions are released from scrapie-infected splenic cell cultures *ex vivo.*

Culture conditions	B cells		T cells		Dendritic cells	
	Release (TCIU/10^6^ cells)	Necrosis (%)	Release (TCIU/10^6^ cells)	Necrosis (%)	Release (TCIU/10^6^ cells)	Necrosis (%)
**atm. CO_2_** †	0.2±0.3	100	<0.1	100	0.2, 0.4	100
**basal**	5.6±1.0	63	1.1±0.3	48	6.8, 10.8	51
**basal+IL4**	6.3±1.2	34	1.9±0.4	22	-	-
**basal+LPS**	4.4±0.8	28	-	-	-.	-
**Cellular infectivity**	227		188		898	

**†:** Control incubations were performed in basal medium at atmospheric (atm.) CO_2_ and 37°C. For dendritic cell cultures Triton X-100 was added to a final concentration of 0.01% in basal medium.

Fifteen 129Sv×C57BL/6 were inoculated i.p. with 100 µl RML I6200 and culled at 60 d.p.i. Splenic cell types were isolated by Collagenase perfusion according to the experimental procedure depicted in Figure 1. The levels of cellular infectivity were determined after MACS isolation. MACS-isolated B and T lymphocytes were then cultured at a concentration of 1×10^6^/ml in basal medium (IMDM medium, 10% FBS) in absence or presence of LPS (50 µg/ml) and IL-4 (10 ng/ml). DCs were cultured in basal medium, supplemented with 200 ng GM-CSF. To remove cells and debris the conditioned medium was collected after 36 h of culture, centrifuged at 300× g for 10 min, 5,000× g for 15 min and 10,000× g for 30 min and the supernatant was collected at each of the sequential centrifugations. The supernatant was then centrifuged for 2 h at 100,000× g and resuspended in PBS, serially diluted and infectious titers were determined using SCEPA. The detection limit for SCEPA was 0.1 TCIU/Mio cells. Mean values ± SE of three independent experiments are shown. Data from two independent experiments are shown for the release of infectivity from DCs.

## Discussion

We characterized the rate of prion accumulation in hematopoietic cells of the spleen at early stages of prion disease and identified highest infectious titers in two cell types that have previously not been associated with prion pathogenesis, pDCs and NK cells. We furthermore report the first experimental evidence for a release of prions from lymphocytes and DCs from scrapie-infected mice *ex vivo*, a process that is associated with the secretion of exosome-like membrane vesicles.

In contrast to the well-defined role of stromal cells during prion colonization in the LRS, the contribution of mobile cells of hematopoietic origin to prion dissemination is not well characterized. Whilst previous studies reported high infectious titers of gradient-enriched cells of low buoyant densities [26], [97] and more specifically of MACS-isolated DCs [25] and lymphocytes [60], data were restricted to single time points and a limited number of cell types. The recent establishment of an *in vitro* infectivity assay, the SCA now enabled us to study the dynamics of prion accumulation in hematopoietic cells of the LRS in a systematic manner. The surge of prions in lymphoid tissues and MACS-isolated cells during the first weeks after inoculation provides evidence for the exceptional rate of prion colonization. In particular, three days after i.p. inoculation, prion titers in spleens of 129Sv×C57/BL6 mice were three orders of magnitude higher than those of prion replication-deficient *Prnp* ^0/0^ mice, implying highly efficient pathways for prion dissemination and replication. A titer of 2.5 log TCIU/g in spleens of *Prnp* ^0/0^, on the other hand is indicative of PrP-independent mechanisms of prion sequestration and dissemination from the site of infection to lymphoid organs. Similar titers were detected in spleen tissue after i.c. inoculation of *Prnp* ^0/0^ mice with RML brain homogenate (2.3 log LD50/ml) [58]. In the absence of prion replication in *Prnp* ^0/0^ mice, infectivity accumulated preferentially in DCs and macrophages and at 5 to 10-fold lower rates in lymphocytes which confirms a role for antigen-presenting cells in prion sequestration [27], [29], [32], [98]–[100].

At 30 dpi, pDCs and NK cells were 7-fold and >2-fold more infectious than lymphocytes, respectively (Table 3 and Figure S2). In agreement with other reports [99] PrP^c^ expression was undetectable in pDCs (Figure S3). Although prion replication-competence of cells cannot be predicted solely on the basis of PrP^c^ expression levels [101], [102], pDCs seemed *a priori* a poor candidate for a role in prion replication. However, that pDCs are instead highly infectious, as shown in this study, underscores the importance of prion sequestration and dissemination by antigen-presenting cells. PDCs are natural type 1 IFN-producing cells, located in the T cell rich periarteriolar lymphoid sheath of lymphoid organs. Their distribution differs from conventional DCs which are predominantly found in the marginal zone and outer PALS, but not in the red pulp of the spleen [103]. Interestingly, in a steady state, NK cells are also found in areas of antigen entry to lymphoid organs, in perifollicular regions, in the paracortex, and especially in the medulla zone within lymphatic sinuses [104]. Whether the distribution of pDCs and NK cells in lymphoid organs is related to their high prion titers has to be further investigated. A bidirectional cross-talk between DCs and NK cells has recently been shown to play a key role in host defense [105], [106]. In contrast to highly infected pDCs, macrophages showed about eight fold lower prion titers. Of note, the *in vivo* depletion of macrophages shortened scrapie incubation times [98], suggesting that macrophages have a protective role on disease progression.

The maturation state of DCs has major implications on antigen processing and cell trafficking and may be critical to better understand the role of DCs in prion pathogenesis. Even though immature DCs are poor in T cell priming, they are efficient in antigen capture and processing [107], [108]. Migration is greatly affected by the maturation state of DCs and immature and activated DCs are recruited by distinct chemokines [107]. Upregulation of CCR7 during maturation renders DCs sensitive to the chemoattractants CCL19/CCL21 [109] and are consequently recruited to T-cell rich areas [107]. Accordingly, mice with a recessive loss of CCL21 and CCL19 expression showed defects in the migration of naïve T cells and activated DCs [110]. When inoculated with mouse prions, however, these mice only showed marginal effects on disease incubation times, indicating that CCL19/CCL21-dependent DC migration to T-cell zones does not seem to contribute to prion accumulation in lymphoid organs [111].

An important study reported a change of tissue tropism of prion accumulation in otherwise non-permissive tissues during experimental inflammatory conditions of the kidney, pancreas, and liver [112]. Follicular inflammatory foci with FDC networks and discrete B220^+^ areas correlated with the propensity of inflamed tissue to replicate prions [112]. Under these conditions, a mobilization of prion-infected immune cells to sites of infection is also likely to transport prions from lymphoid to affected organs.

Under certain neurological conditions DCs are recruited into the CNS. PDCs, for example are the major CNS-infiltrating cells during experimental autoimmune encephalomyelitis (EAE) [113]. Of note, prion disease progression was accelerated by induction of EAE in scrapie infected mice [114].

Despite a rapid increase of prion titers in the LRS at early stages of disease, prions were only detected at extremely low titers in blood of presymptomatic and symptomatic animals [63]–[68]. Four weeks after inoculation DCs and pDCs in blood did not contain detectable infectivity. The limited capacity of DCs for recirculation [69] may greatly restrict the dissemination of prions through the hematogenous route. Recirculation of NK cells is also restricted under steady-state conditions [115]. While restricted recirculation of DCs may protect the host by retaining a high density of particular peptide-MHC complexes for improved antigen presentation [69], inflammatory signals induce tissue-resident DCs to undergo maturation and to migrate into inflamed tissues [116].

Our evidence for a release of prions from scrapie-infected DCs and lymphocytes suggests a potential route for the lateral spread of prions and may contribute to the striking rate of prion colonization in the LRS. Antigen-presenting cells, like DCs, macrophages and B cells are specialized to phagocytose pathogens and to present processed antigen, loaded onto MHC class II molecules to T lymphocytes. While it is a matter of debate whether exosomes bearing MHC class II peptide complexes actively support the immune response of the host [84], [117]–[119], the dissemination of pathogens via exosomes is not a novel concept. Retroviruses were shown to redirect the cellular protein sorting machinery to egress infected cells at the level of the plasma membrane and to usurp the existing cellular machinery for exosomal release, respectively (for recent reviews see [120]–[123]). Of note, the release of prion-infected exosomes was enhanced by retroviral infection [124], suggesting the existence of synergistic mechanisms during endosomal processing. Irrespective of their sites of conversion, prions will reach the endosomal route, a cellular pathway that renders prion-infected lymphocytes and DCs at risk for a lateral spread of prions. A segregation of prions into the exosomal route would enable a transfer of infectivity between cells without direct cell-to-cell contact. Of note, B cell-derived exosomes bind preferentially to surface receptors on FDCs [44]. Exosome release as a potential dissemination route has also been suggested for other misfolded proteins, like Aβ peptides in Alzheimer's disease andv α-synuclein in Parkinson's disease and dementia with Levy bodies, respectively [125], [126].

## Materials and Methods

### Ethics statement

All animal experiments were performed in compliance with United Kingdom Home Office regulations and were approved by both the Home Office and the MRC Prion Unit ethical review committee.

### Mice and scrapie infection

Six to eight week old female 129/Sv×C57BL/6 mice were purchased from Harlan UK Ltd. (Oxfordshire, UK). *Prnp* ^0/0^ mice used here were derived from the original Zurich I mice [127] and crossed onto the FVB/N background for 10 generations [128]. Mice were inoculated intraperitoneally (i.p.) with 100 µl of 1% Rocky Mountain Laboratory (RML) prion strain I6200 [38] or 1% uninfected CD1 brain homogenate and culled at early stages of prion disease prior to the manifestation of neurological symptoms. Where prion titers were determined by mouse bioassay, mice were inoculated intracerebrally (i.c.) with 30 µl inoculum and the incubation time until manifestation of neurological signs of scrapie was recorded. All mice were observed daily for indications of ill-health.

### Isolation of splenocytes

Splenocytes were isolated by enzymatic digestion from freshly dissected spleens. To maximize the release of non-haematopoietic stromal cells and other resident cells that are strongly attached to connective tissue, spleens were digested in successive cycles as described previously with minor modifications [129]. Briefly, spleens were cut into small pieces and incubated at 37°C with an enzyme cocktail, containing 2.5 mg/ml collagenase IV (Worthington Biochemical Corp., Lakewook, NJ), 0.05% dispase 2 (Sigma-Aldrich, UK) and 1 mg/ml DNase I (Roche Diagnostics Limited, West Sussex, UK) in Iscove's Modified Dulbecco's Media (Invitrogen, Paisley, UK), supplemented with 10% heat-inactivated FBS, 100 U/ml Pen-strep, 2 mM L-glutamine and 50 µM 2-mercaptoethanol (complete IMDM) per spleen. After 15–20 min, partially digested tissue was gently dispersed with a serological pipette and released cells were transferred into a tube on ice. Fresh enzyme cocktail was added to the remaining tissue fragments and digested for another three cycles. Pooled cells were passed through a 70 µm nylon mesh and pelleted at 300× g for 10 min. To remove erythrocytes splenocytes were resuspended in 10 ml erythrocyte lysis buffer (155 mM NH_4_Cl, 10 mM KHCO_3_, 0.1 mM EDTA, pH 7.0) and incubated at room temperature for no more than 1 min. After adding 40 ml complete IMDM medium to stop lysis cells were pelleted. Splenocytes were then layered onto Lympholyte M (Cedarlane Laboratories, Hornby, Ontario, Canada) gradients and centrifuged at 1500× *g* for 20 min to remove dead cells and debris essentially as described by the manufacturer. Purified splenocytes were washed in complete IMDM and centrifuged for 10 min at 800× *g*. Cells were resuspended in chilled MACS buffer (0.5% bovine serum albumin (BSA) and 2 mM EDTA in phosphate-buffered saline) and the number of splenocytes was determined using a Coulter counter Z2 (Beckman Coulter) at an upper threshold of 15 and a lower threshold of 5.

### Isolation of splenic cell types by magnetic-activated cell sorting

Specific cell populations were enriched from total splenocytes by sequential MACS using antibody-coated magnetic beads (Miltenyi Biotech Ltd., Surrey, UK) as depicted in Figure 1. To block unwanted binding of antibodies to cells expressing Fc receptors (FcR) splenocytes were suspended at a concentration 2×10^8^ cells/ml MACS buffer and incubated with 25 µl FcR blocking reagent (Miltenyi) per 10^8^ cells. Cells were magnetically labelled essentially as specified by the manufacturer (Miltenyi) using the following microbeads: CD11c for DCs, mPDCA-1 for pDCs, CD11b for myeloid cells, CD49b for NK, CD19 for B cells and CD90 for T cells. Splenic DCs comprise three distinct subsets of CD11c^+^ cells, CD11c^+^ CD11b^+^ myeloid DCs, CD11c^+^ CD8^+^ lymphoid DCs and CD11c^low^ CD45R (B220^+^) pDCs. To avoid a loss of pDCs which express low levels of CD11c during panDC isolation a combination of CD11c and murine plasmacytoid dendritic cell antigen-1 (mPDCA-1) beads was used. In murine spleen, bone-marrow and lymph nodes, mPDCA-1 is exclusively expressed on interferon-producing cells which are CD11c^+^ CD45R (B220^+^) Ly-6C^+^ [130]. Positive selection of CD11c^+^ dendritic cells prior to isolating CD11b^+^ macrophages did not suffice to deplete CD11c^+^CD11b^+^ myeloid dendritic contaminants (Figure 1). We therefore purified macrophages by fluorescence-activated cell sorting (FACS) using a MoFlo cell sorter (Dako). Briefly, MACS-isolated CD11b^+^ cells were incubated with FITC-conjugated anti-CD11c (clone HL3, 1∶100) and PE-conjugated anti-CD11b (clone M1/70, 1∶50) (BD Biosciences, Oxford, UK) and CD11c^−^ CD11b^+^ cells were sorted at a concentration of 5–10×10^6^ cells per ml. Isolated cells were counted with a Coulter Counter, snap-frozen in liquid N_2_ and stored at −80°C until further processing. The purity of isolated cell types was determined by FACS using a FACS calibur (BD Bioscience).

### Analytical flow cytometry

Isolated cell types were characterized by flow cytometry using the following fluorescent-conjugated mAbs: anti-B220/CD45R (RA3-6B), anti-CD90/Thy1.2 (30-H12), anti-CD11c (HL3), anti-CD49b (DX5), anti-CD21/CD35 (76G), anti-CD23/FcεRII (B3B4) and anti-CD86 (GL1) were purchased from BD Pharmingen (Oxford, UK). Anti-CD11b (M1/70) and anti-CD49b (DX5) were purchased from eBioscience (Hatfield, UK). All fluorescence- or biotin-conjugated isotype controls (rat IgG_2b_, rat IgG_2a_, Armenian hamster IgG_1_, mouse IgG_1_, κ, rat IgG M) were purchased from eBioscience. Briefly, aliquots of 1–2×10^6^ cells were resuspended in MACS buffer, incubated for 15 min with FcR blocking reagent (1∶20, Miltenyi) on ice and labeled with fluorescent-conjugated antibodies or isotype controls for 30 min. Data acquisition and analysis was performed using a FACS calibur and CellQuest software (BD Biosciences).

### Isolation of lymphocytes and pDCs from blood

Whole blood was obtained from euthanized mice by cardiac puncture and collected in buffered EDTA-containing syringes with a final EDTA concentration of 2 M. Blood samples were diluted one in four into MACS buffer, layered onto Lympholyte M and centrifuged for 20 min at 1500× *g* at 22°C. Cells from the interface were collected and erythrocytes removed as described before. After washing, blood cells were resuspended in MACS buffer and pDCs and lymphocytes were isolated by MACS as described above. To check the efficacy of cell capture from blood samples by MACS whole blood was spiked with 2 Mio prion-infected B lymphocytes (44 TCIU) and cells were isolated as described above. A 82% (36 TCIU) recovery of infectious B lymphocytes confirms the excellent performance of MACS isolation from blood samples.

### Isolation of exosome-enriched membrane fractions and analysis by electron microscopy

Exosomes were isolated by differential centrifugations described as previously [82]–[84]. Briefly, supernatants from cell cultures of splenic cell types were retrieved after 38 h and sequentially centrifuged at 300× g for 10 min, 5,000× g for 20 min and 10,000× g for 30 min, and finally at 100,000× g for 2 h. Pellets were resuspended in PBS and used immediately or stored at −70°C until further use. For analysis by electron microscopy 3 µl aliquots of 1∶10 dilutions of resuspended pellets were adsorbed onto glow-discharged carbon-coated grids and negatively stained with 1% uranyl acetate. Grids were examined by electron microscopy at the Bloomsbury Centre for Structural Biology (Birkbeck College, London, UK). To determine the number of exosome-like membrane particles 20 random images were recorded per condition and the number of particles was counted in a blinded manner.

### Immunoisolation of exosomes

Exosomes from cultured B lymphocytes or splenocytes were enriched with antibodies against exosome markers Rab 5B [96] and CD81 [93]–[95] using a μMACS streptavidin kit (Miltenyi). Briefly, concentrated cell culture supernatants of 5×10^7^ cells were obtained by differential centrifugation as described above, resuspended in 150 µl PBS and incubated for 30 min with 10 µg biotinylated antibodies anti-CD81 (clone Eat-2, Biolegend), anti-Rab 5B (clone A 20, Santa Cruz Biotechnology) or isotype controls (rabbit and Armenian hamster IgG, eBioscience). After addition of 100 µl μMACS beads immune complexes were incubated for 10 min and captured on μMACS columns according to the specifications of the manufacturer. Infectious titers of immuno-isolated fractions were determined by SCEPA.

### Preparation of the RML standard dilutions

RML standard dilutions used for *in vitro* and *in vivo* infectivity assays were prepared by serial 10-fold dilutions (from 10^−2^ to 10^−9^) of 10% RML homogenates into 10% uninfected CD1 brain homogenate. Diluted brain homogenates were further diluted 1∶10 into 1% normal CD1 for inoculation into Tg20 mice and 1∶1000 into OFCS for infection of cells, respectively.

### Preparation of tissue and cell homogenates

To determine infectious titers of tissue samples spleens and mesenteric lymph nodes from scrapie-infected and control mice were minced and transferred into 2 ml microtubes (Sarstedt Ltd., Leicester, UK) containing zirconium beads. Ten percent homogenates (w/v) were prepared in PBS-buffered sucrose (0.32 M) in presence of a 1∶100 dilution of Protease Inhibitor Cocktail Set I (Pierce, Leicestershire, UK) and 25–50 U benzonase (Novagen, Madison, WI) using a Ribolyser (Hybaid, Cambridge, UK) at maximum speed for two cycles of 45 s. Aliquots of tissue homogenates were serially diluted 1∶10 into 10% uninfected CD1 brain homogenate (w/v) to minimize binding to surfaces and stored at −80°C until infectious titers were determined *in vitro* and on mouse bioassay, respectively.

Aliquots of MACS-isolated splenic cells were ribolyzed at a concentration of typically 2×10^7^ cells/ml complete medium, supplemented with protease inhibitors as described above. All homogenates were kept on ice until further processing.

Where sonication was used to homogenize cells, aliquots of MACS-isolated cells were transferred into 0.2 ml Thermo tubes (Thermo Fisher Scientific, West Sussex, UK) and placed beneath the sonication probe in ice water. Cells were homogenized in five cycles of 30 s at 30% power using a Status 200 sonicator (Philip Harris Scientific, Hyde, UK). Brain homogenates were prepared by repeated passing through syringe needles as described elsewhere [131].

### Quantification of prion titers by Scrapie Cell Assay and mouse bioassay

Infectious titers were determined *in vitro* by Scrapie Cell Assay in endpoint format (SCEPA) as described previously [36], [52] with minor modifications. Briefly, 2×10^4^ PK1-2 cells in Opti-MEM-10% FCS (OFCS) were plated into wells of 96-well plates. After 16 h cells were incubated with 300 µl aliquots of serially diluted homogenates. Three days later cells were initially split twice 1∶2 every other day and 1∶3 two days after the second split. Prior to resuspending cells half the medium was replaced with fresh OFCS for all previous cell passages. After three days cells were split 1∶6 every 3–4 d. Aliquots of 25,000 cells were transferred onto Elispot plates after the sixth and seventh split (MultiScreen HTS-IP Filter Plate, Millipore) and the number of PrP^Sc^-positive cells was determined by ELISA after incubation with 4.4 mU (1 µg) recombinant PK (Roche Diagnostics, West Sussex, UK) per ml lysis buffer as described previously [36]. The sensitivity of SCEPA was determined from serial dilutions of titered mouse RML brain homogenate I6200 (9.3 log LD50 units/g brain). Infectious titers obtained *in vitro* were expressed as tissue-culture infectious units (TCIU). Mouse bioassay were performed by intracerebral inoculation of groups of six Tga20 mice [132] with 30 µl of serially diluted samples.

### Detection of protease K-resistant PrP

To determine the levels of protease K (PK)-resistant PrP 10% spleen homogenates were prepared by ribolyzing freshly dissected spleens in PBS-buffered sucrose (0.32 M) in presence of protease inhibitors as described above. The levels of PK-resistant PrP were determined by Western Blotting after precipitation of PrP with sodium phosphotungstic acid (NaPTA) as described previously [133].

### Immunohistochemistry

Spleens were fixed in 10% buffered formal saline for 24 h prior to tissue processing and paraffin wax embedded. All spleen samples were coded prior to sectioning and histological analysis of spleens sections was carried out blinded. Sections were cut at a nominal thickness of 4 µm, and stained with hematoxylin and eosin using conventional methods. To detect abnormal PrP deposition, mounted sections were placed on a Ventana automated immunohistochemical staining machine (Ventana Medical Systems, Tuscon, AZ, USA), heated to 95°C in a proprietary buffer, for 90 minutes (Ventana Medical Systems), incubated in Superblock for 10 minutes, then exposed to biotinylated ICSM35 (1∶25 dilution of 1.6 µg/mL stock; D-Gen Ltd, London, UK), followed by an avidin-biotin horseradish peroxidase conjugate (DABmap, Ventana Medical Systems) and developed with 3-3′-diaminobenzidine tetrahydrochloride. Hematoxylin was used as counterstain. Appropriate controls were used throughout. Photographs were taken using the slide scanner LEICA SCN400 (LEICA Microsystems).

## Supporting Information

Figure S1 **Initial validation of Poisson distribution.** To check whether the experimental data from in-vitro endpoint titrations indicate an underlying Poisson distribution for the number of infected cells serial 1∶3 dilutions of RML brain homogenate within a range between 10-7 and 10-9 were prepared and cell layers of 12 wells per dilution were infected. Complementary log-log transformed proportions of negative wells are shown for eight technical assay repeats. Linear regression analysis was performed for dilutions were the proportion of positive wells for all eight repeats were >0 and <12 per total number of wells and a slope factor β of 1.06±0.20 was calculated.
(TIF)

Figure S2 **Differences in prion accumulation in splenic cells at 30 dpi.** Infectious titers of MACS-isolated splenic cell types at 30 dpi were replotted from Table 3 for clarity. Significant differences between distinct cell types and lymphocytes are indicated (*p<0.05; ** p<0.001). Infectious titers of the CD11b+ myeloid cells decreased by about 50% after FACS purification of CD11c− D11b+ macrophages (see Fig. 1).
(TIF)

Figure S3 **Protein expression levels of PrPc are undetectable in pDCs of 129Sv×C57BL/6 mice.** PDCs isolated from uninfected 129Sv×C57BL/6 or Prnp−/− mice were labeled with biotinylated monoclonal anti-PrP antibody ICSM 35 followed by allophycocyanin (APC)-streptavidin and PrPc expression levels were analysed by flow cytometry. No difference in PrPc expression levels between pDCs from 129Sv×C57BL/6 (wildtype) and Prnp−/− mice was detected. As a control for PrPc expression mouse neuroblastoma cells (N2a) were labeled with biotinylated ICSM35 and biotinylated mouse IgG2b isotype control.
(TIF)

Figure S4 **DCs are not activated at preclinical stages of Scrapie.** 129Sv×C57BL/6 mice were inoculated i.p. with 100 µl 1% RML I6200 or 100 µl 1% uninfected CD1 homogenate and splenocytes were isolated at 110 dpi. DCs were isolated by MACS using a 1∶1 mix of CD11 and mPDCA-1 microbeads and labelled with a specific mAb against CD86. No evidence for an expression difference of CD86 between scrapie-infected and age-matched control mice were detected at preclinical stages.
(TIF)

Figure S5 **No abnormalities of splenic B cell subsets at preclinical disease.** 29Sv×C57BL/6 mice were inoculated i.p. with 100 µl 1% RML I6200 or 100 µl 1% uninfected CD1 homogenate and culled at 80 dpi (A) and 100 dpi (B). Splenocytes were isolated according to Materials and Methods. To analyse B cell subsets splenocytes were labelled with mAbs against anti-CD19, anti-CD23 and anti-CD21. The CD19-gated B cell population was examined for CD21/35 and CD23 expression. No alterations were detected in the ratios of CD21high D23- marginal zone (MZ) and CD21int D23high follicular (FO) B cells.
(TIF)

Table S1 **In-vitro endpoint titration of RML 6200 using SCEPA.** Serially diluted RML 6200 was transferred onto layers of prion-susceptible PK1 cells and the number of positive and negative wells was determined by SCEPA as described in Materials and Methods. The complementary log-log transformed data were plotted in Figure S1.
(RTF)

Table S2 **Immunohistological characterisation of scrapie-infected spleen tissue at early stages after infection.** An increase in the number of lymphoid follicles containing follicular dendritic cells containing abnormal prion protein (ICSM35 immunostaining) as well as an increase in the density of PrPSc deposition is seen with increasing incubation time. Positive follicles were determined as the ratio of the number of ICSM35-positive follicles and the total number of follicles (counted on an adjacent H&E section. PrPSc density in follicles was determined semi-quantitatively as weak (shown in Figure 3C, 3 and 7 dpi), moderate (Figure 3C, 14 and 30 dpi) and strong.
(RTF)

Table S3 **Infectious titers of MACS-isolated splenic cell types in the absence of prion replication at 3 dpi.** A group of four Prnp−/− mice were inoculated intraperitoneally with 100 µl 1% (w/v) RML I6200 and spleens were dissected at 3 dpi. Different cell types were isolated by magnetic sorting, infectious titers determined by SCEPA and titers estimated by GLM.
(RTF)

Text S1 **Determination of infectious titers from SCEPA using GLM regression.**
(RTF)
