# Supplementary figures and images for: Plasmacytoid Dendritic Cells Sequester High Prion Titres at Early Stages of Prion Infection
Source: PLoS Pathog. 2012 Feb 16;8(2):e1002538. doi: 10.1371/journal.ppat.1002538 (PMC3280992; doi:10.1371/journal.ppat.1002538)

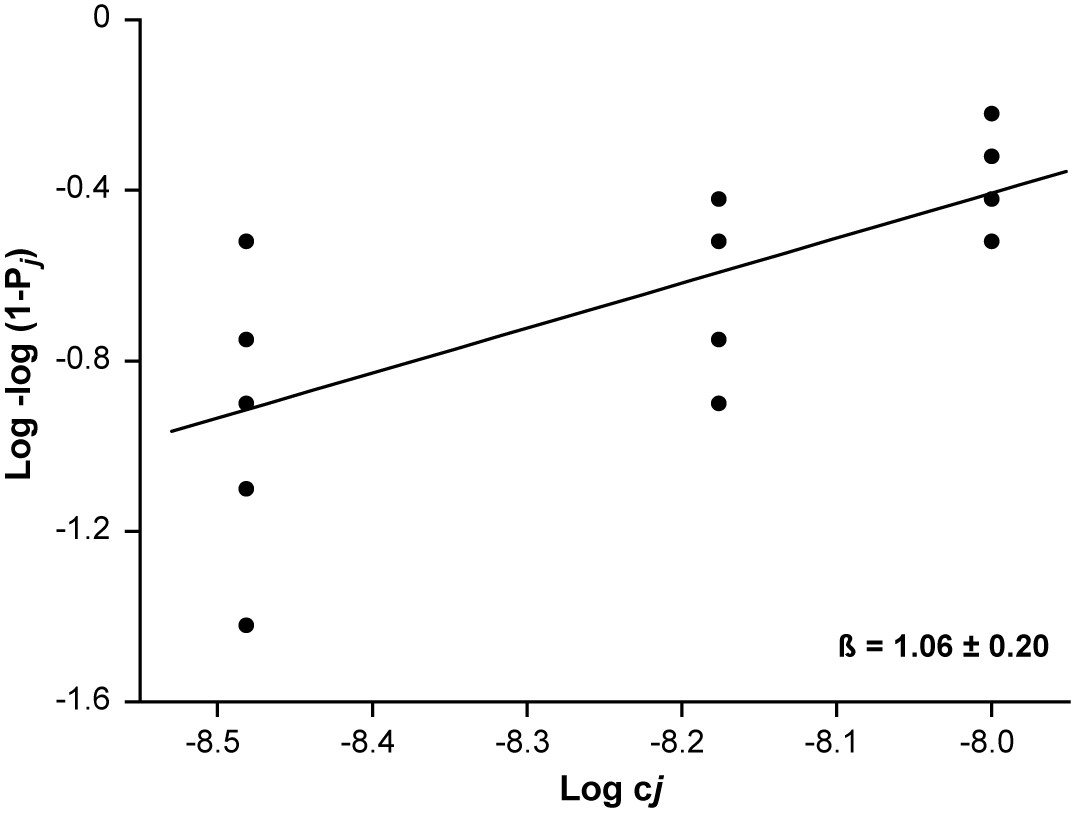

Supplement: Figure S1 — Initial validation of Poisson distribution. To check whether the experimental data from in-vitro endpoint titrations indicate an underlying Poisson distribution for the number of infected cells serial 1∶3 dilutions of RML brain homogenate within a range between 10-7 and 10-9 were prepared and cell layers of 12 wells per dilution were infected. Complementary log-log transformed proportions of negative wells are shown for eight technical assay repeats. Linear regression analysis was performed for dilutions were the proportion of positive wells for all eight repeats were >0 and <12 per total number of wells and a slope factor β of 1.06±0.20 was calculated. (TIF) [file ppat.1002538.s001.tif]

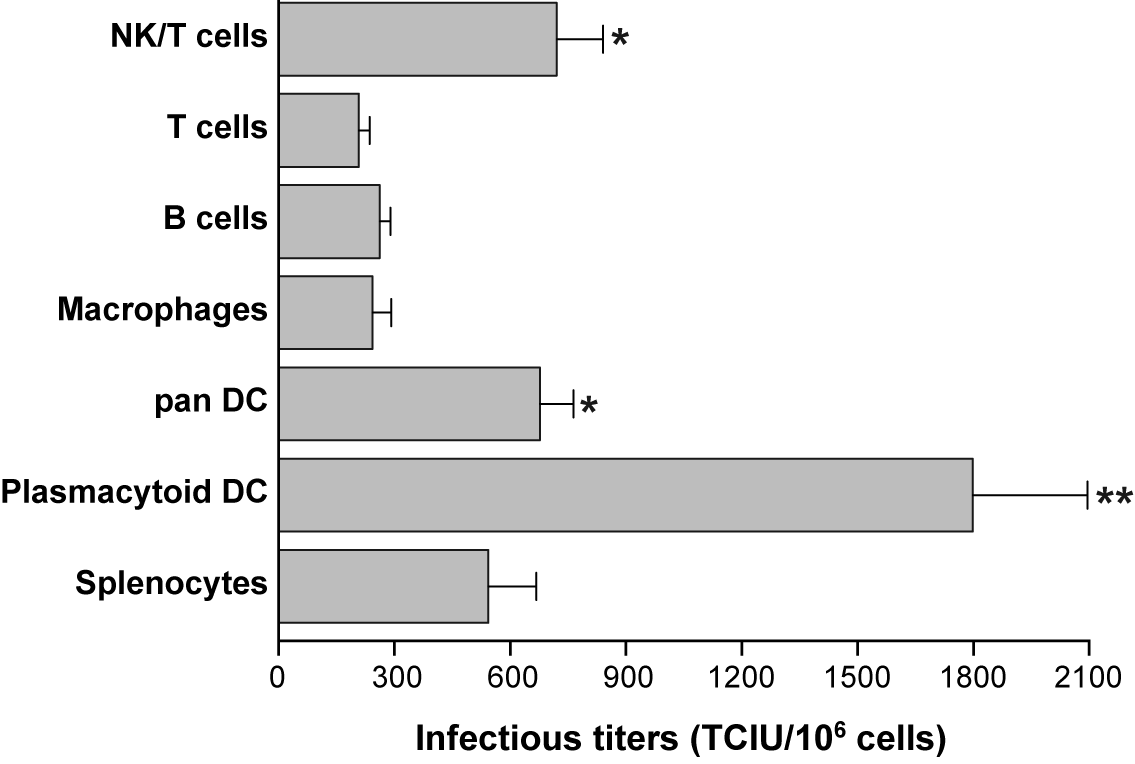

Supplement: Figure S2 — Differences in prion accumulation in splenic cells at 30 dpi. Infectious titers of MACS-isolated splenic cell types at 30 dpi were replotted from Table 3 for clarity. Significant differences between distinct cell types and lymphocytes are indicated (*p<0.05; ** p<0.001). Infectious titers of the CD11b+ myeloid cells decreased by about 50% after FACS purification of CD11c− D11b+ macrophages (see Fig. 1). (TIF) [file ppat.1002538.s002.tif]

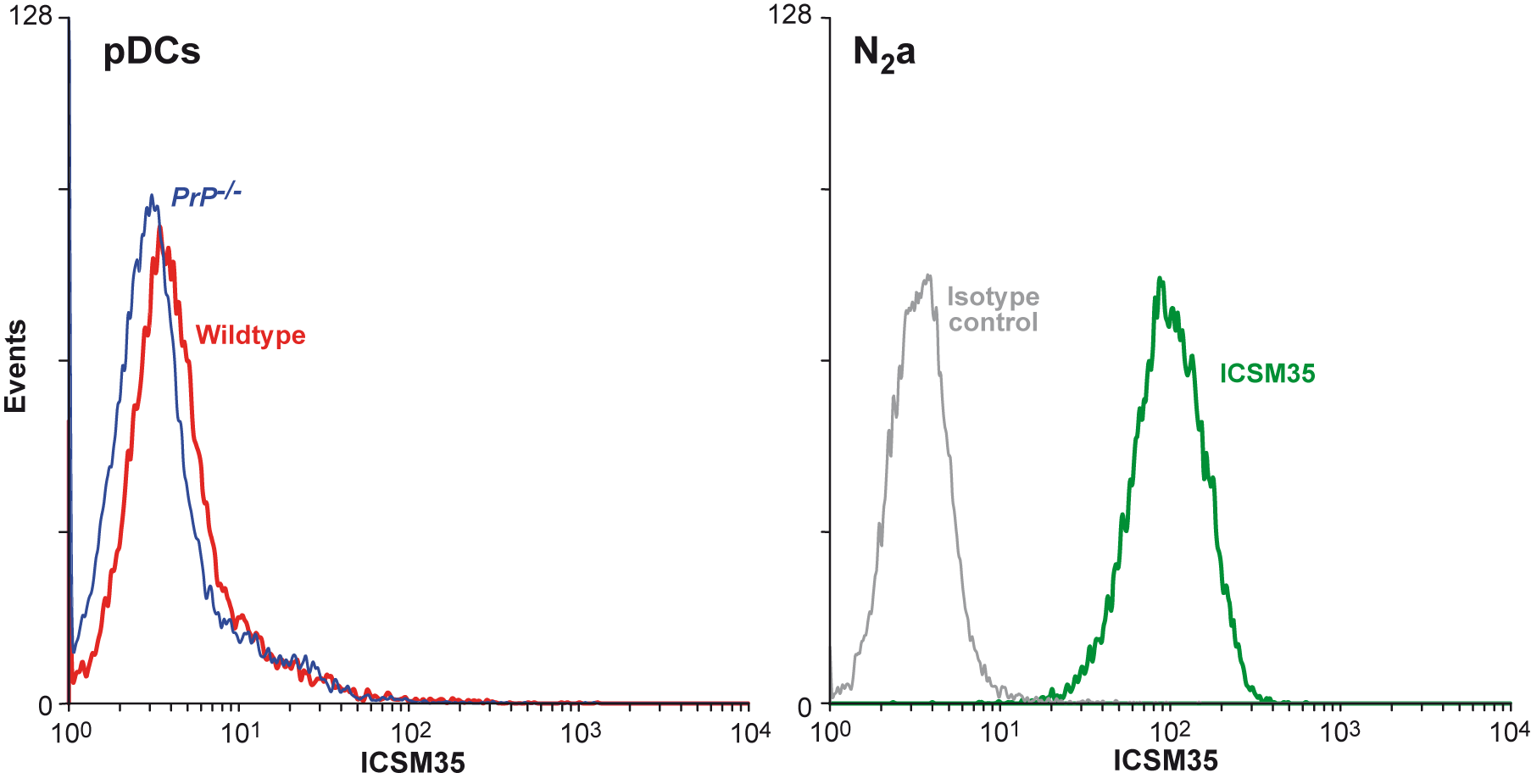

Supplement: Figure S3 — Protein expression levels of PrPc are undetectable in pDCs of 129Sv×C57BL/6 mice. PDCs isolated from uninfected 129Sv×C57BL/6 or Prnp−/− mice were labeled with biotinylated monoclonal anti-PrP antibody ICSM 35 followed by allophycocyanin (APC)-streptavidin and PrPc expression levels were analysed by flow cytometry. No difference in PrPc expression levels between pDCs from 129Sv×C57BL/6 (wildtype) and Prnp−/− mice was detected. As a control for PrPc expression mouse neuroblastoma cells (N2a) were labeled with biotinylated ICSM35 and biotinylated mouse IgG2b isotype control. (TIF) [file ppat.1002538.s003.tif]

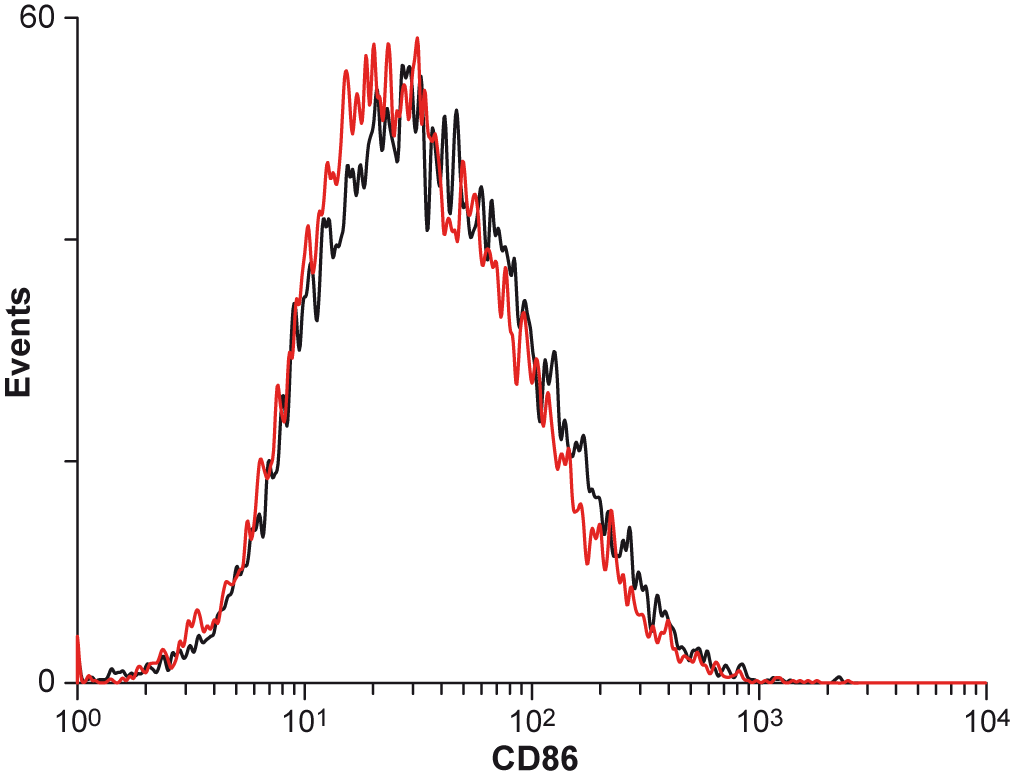

Supplement: Figure S4 — DCs are not activated at preclinical stages of Scrapie. 129Sv×C57BL/6 mice were inoculated i.p. with 100 µl 1% RML I6200 or 100 µl 1% uninfected CD1 homogenate and splenocytes were isolated at 110 dpi. DCs were isolated by MACS using a 1∶1 mix of CD11 and mPDCA-1 microbeads and labelled with a specific mAb against CD86. No evidence for an expression difference of CD86 between scrapie-infected and age-matched control mice were detected at preclinical stages. (TIF) [file ppat.1002538.s004.tif]

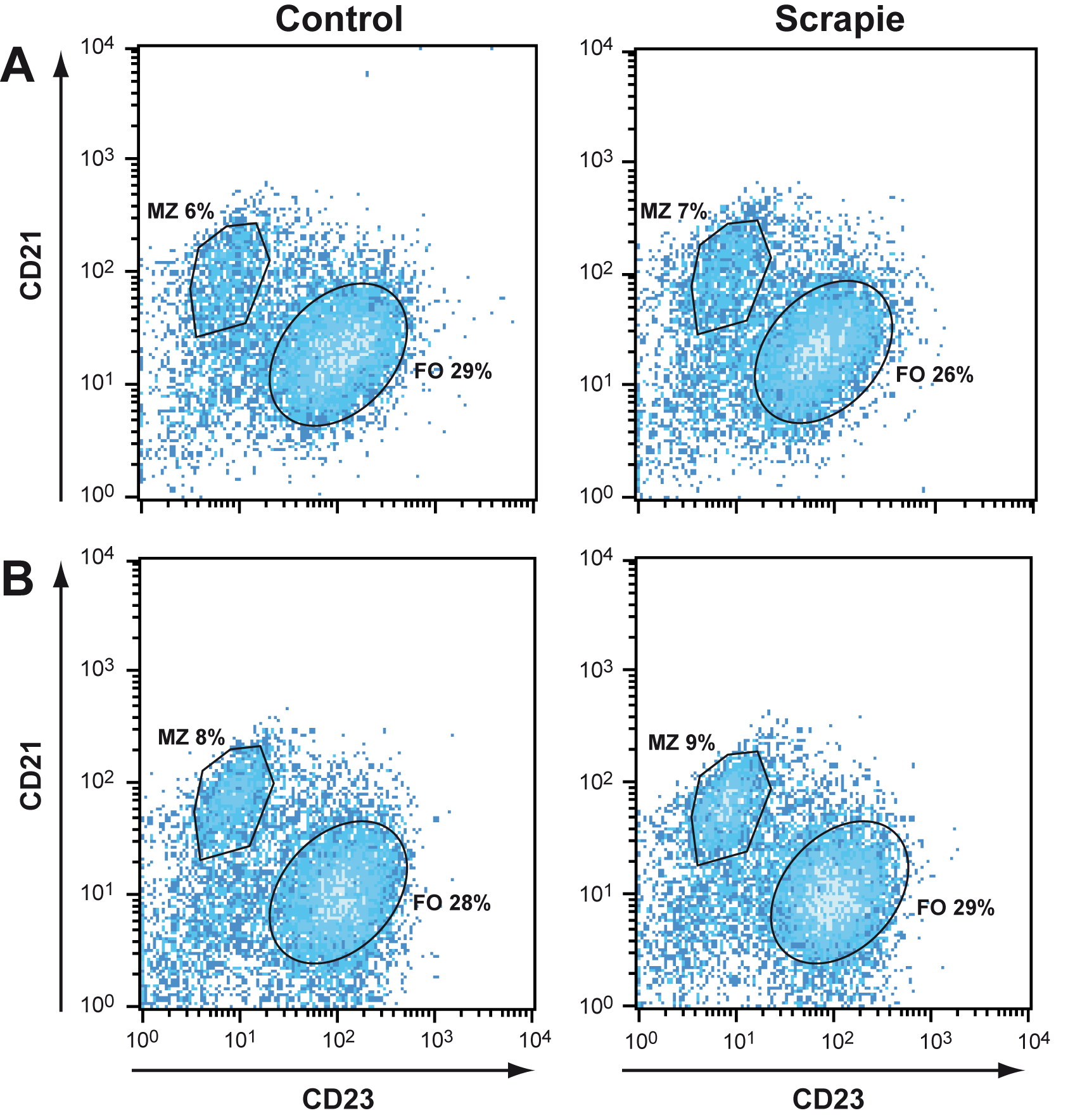

Supplement: Figure S5 — No abnormalities of splenic B cell subsets at preclinical disease. 29Sv×C57BL/6 mice were inoculated i.p. with 100 µl 1% RML I6200 or 100 µl 1% uninfected CD1 homogenate and culled at 80 dpi (A) and 100 dpi (B). Splenocytes were isolated according to Materials and Methods. To analyse B cell subsets splenocytes were labelled with mAbs against anti-CD19, anti-CD23 and anti-CD21. The CD19-gated B cell population was examined for CD21/35 and CD23 expression. No alterations were detected in the ratios of CD21high D23- marginal zone (MZ) and CD21int D23high follicular (FO) B cells. (TIF) [file ppat.1002538.s005.tif]
