# Supplementary material for: Plasmacytoid Dendritic Cells Sequester High Prion Titres at Early Stages of Prion Infection
Source: PLoS Pathog. 2012 Feb 16;8(2):e1002538. doi: 10.1371/journal.ppat.1002538 (PMC3280992; doi:10.1371/journal.ppat.1002538)
Supplement: Table S1 — In-vitro endpoint titration of RML 6200 using SCEPA. Serially diluted RML 6200 was transferred onto layers of prion-susceptible PK1 cells and the number of positive and negative wells was determined by SCEPA as described in Materials and Methods. The complementary log-log transformed data were plotted in Figure S1. (RTF) [file ppat.1002538.s006.rtf]

Table S1: In-vitro endpoint titration of RML 6200 using SCEPA.

		Independent SCEPAs	
Dilution	Wells	1	2	3	4	5	6	7	8	
3.3x10^-8^	positive	11	12	12	12	11	10	12	10	
	negative	1	0	0	0	1	2	0	2	
10-8	positive	9	6	8	9	7	8	8	6	
	negative	3	6	4	3	5	4	4	6	
6.6x10^-9^	positive	6	4	4	4	3	7	6	4	
	negative	6	8	8	8	9	5	6	8	
3.3x10^-9^	positive	4	6	2	4	1	4	3	3	
	negative	8	6	10	8	11	8	9	9	
