# Supplementary material for: Plasmacytoid Dendritic Cells Sequester High Prion Titres at Early Stages of Prion Infection
Source: PLoS Pathog. 2012 Feb 16;8(2):e1002538. doi: 10.1371/journal.ppat.1002538 (PMC3280992; doi:10.1371/journal.ppat.1002538)
Supplement: Table S2 — Immunohistological characterisation of scrapie-infected spleen tissue at early stages after infection. An increase in the number of lymphoid follicles containing follicular dendritic cells containing abnormal prion protein (ICSM35 immunostaining) as well as an increase in the density of PrPSc deposition is seen with increasing incubation time. Positive follicles were determined as the ratio of the number of ICSM35-positive follicles and the total number of follicles (counted on an adjacent H&E section. PrPSc density in follicles was determined semi-quantitatively as weak (shown in Figure 3C, 3 and 7 dpi), moderate (Figure 3C, 14 and 30 dpi) and strong. (RTF) [file ppat.1002538.s007.rtf]

Table S2: Immunohistological characterisation of scrapie-infected spleen tissue at early stages after infection.


Incubation time [dpi]	Positive follicles [%]	PrPSc staining intensity (number of mice)	
	Mean ± Stdv		
3
7
14
30	4.5 ± 4.8
36.0 ± 11.5 **
37.5 ± 8.1
91.0 ± 11.9	weak (4)
weak (3)
weak (2), moderate (2)
moderate (3), strong (1)	

** Difference positive follicles 3 dpi versus 7 dpi: p < 0.01
