# Supplementary material for: Plasmacytoid Dendritic Cells Sequester High Prion Titres at Early Stages of Prion Infection
Source: PLoS Pathog. 2012 Feb 16;8(2):e1002538. doi: 10.1371/journal.ppat.1002538 (PMC3280992; doi:10.1371/journal.ppat.1002538)
Supplement: Table S3 — Infectious titers of MACS-isolated splenic cell types in the absence of prion replication at 3 dpi. A group of four Prnp−/− mice were inoculated intraperitoneally with 100 µl 1% (w/v) RML I6200 and spleens were dissected at 3 dpi. Different cell types were isolated by magnetic sorting, infectious titers determined by SCEPA and titers estimated by GLM. (RTF) [file ppat.1002538.s008.rtf]

Table S3: Infectious titers of MACS-isolated splenic cell types in the absence of prion replication at 3 dpi.

Cell types	TCIU/106 cells ± SE#	
pan DC	0.23 ± 0.09	
macrophages	0.47 ± 0.17	
B cells	0.06 ± 0.06	
T cells	0.06 ± 0.06	

# Infectious titers were calculated using a GLM with binomial family complementary log-log link.
