# Supplementary material for: Plasmacytoid Dendritic Cells Sequester High Prion Titres at Early Stages of Prion Infection
Source: PLoS Pathog. 2012 Feb 16;8(2):e1002538. doi: 10.1371/journal.ppat.1002538 (PMC3280992; doi:10.1371/journal.ppat.1002538)
Supplement: Text S1 — Determination of infectious titers from SCEPA using GLM regression. (RTF) [file ppat.1002538.s009.rtf]

Text S1: Determination of infectious titers from SCEPA using GLM regression
GLMs constitute a class of statistical models that relate observations to linear combinations of predictor variables. GLM regression can be applied to a wide range of probability distributions that belong to the 'exponential family' (e.g. normal, binomial, Poisson, and gamma distribution) by the use of a common method for computing maximum likelihood estimates. In a standard linear model , the expected value of the jth response  is equal to a linear combination of explanatory variables  termed the linear predictor   , thus:

 		(1)

In a GLM the link function g, a monotone differentiable function, defines the relationship between the linear predictor ç and the mean of the distribution, thus:

						(2)

The inverse of the link function is   . For prion titer determinations by endpoint assays the response variable , the number of positive wells at the jth dilution, is linked to the dilution by a complementary log-log transformation  , where   and  is the number of independent infections at the jth dilution, via

				(3)

where    is the jth log dilution, á is the log mean number of infectious units and â is the slope parameter.  Other link functions for the binomial model used for titer determination by bioassay are the logit function   and the probit function  . The probit function is the inverse cumulative distribution function of the standard normal distribution. These three common link functions and their inverses are summarised below:

			
Complementary log-log			
Logit			
Probit			


The complementary log-log transformation  arises naturally for in-vitro titres since if the number of infected cells follows a Poisson distribution then the proportion   of negative wells at dilution   is equal to , where m is the mean number of infectious units per volume, which is equivalent to relation (3) with   . 

The parameters á and â are estimated by the method of maximum likelihood. For a given probability distribution specified by   and observations   the log likelihood function for è is

 			(4)

where   is the likelihood function.

R code for the determination of infectious titers by GLM regression
The observed proportions of negative wells from multiple serial dilution of RML (supplementary Table S1) can be fitted using a GLM with binomial family complementary log-log link by the R function:

fit <-glm(cbind(positive,negative)~logdil, data = ori, family=binomial (link="cloglog"))


This code fits a 'saturated' model in which the slope parameter b is arbitrary. The 'reduced' model with a unit slope, corresponding to a Poisson model for the number of infected cells, can be fitted using the following R function:

fit <-glm(cbind(positive,negative)~1, data = ori, offset=logdil, family=binomial (link="cloglog"))

To check the fit of a GLM with binomial family and complementary log-log link we can use the analysis of deviance, a method to test whether the discrepancies between the observed and predicted values lie within an acceptable limit of experimental error.


	Saturated model	Reduced model	
Slope	0.9608	1	
Standard error	0.0969	-	
Degrees of freedom	30	31	
Residual deviance	26.82	26.99	
AIC	106.88	105.04	

The estimated value for the slope factor b is 0.961 ± 0.097. This allows us to test the null hypothesis that the true slope is one using the Wald test. The p-value corresponding to the z-score (0.9608  - 1)/ 0.0969 is 0.69, indicating no evidence of a departure from an underlying Poisson distribution for the number of infected cells.  Alternatively we can test this hypothesis using the likelihood ratio test statistic (LRS)


Where  and  are the maximized likelihoods under the saturated and reduced models, respectively, and   and   are the respective deviances. The LRS approximately follows a Chi-square distribution with degrees freedom equal to the difference in the number of parameters fitted in the two models, here one.  Here LRS = 26.99 - 26.82 = 0.17, giving a p-value of 0.68, approximately the same as the p-value from the Wald test.  The reduction of AIC is a further indication of the superiority of the reduced model.

In both the saturated and reduced models, the residual deviance, which is a measure of how much the data deviates from the model, is lower than the residual degrees of freedom, indicating that the data are not overdispersed in relation to an underlying binomial distribution. The reduced model yielded an estimated titer of 8.63 ± 0.03 logTCIU/g brain for eight technical repeats of serially diluted RML I6200 brain homogenate.

The complete R code for the estimation of infectious titers can be supplied on request.
